# Environmental and mineralogical studies on the stream sediments of Baltim–El Burullus coastal plain, North Delta, Egypt

**DOI:** 10.1038/s41598-024-54045-5

**Published:** 2024-02-15

**Authors:** A. M. Sallam, A. A. Faheim, Z. A. El-Elshafiey, M. M. Abdel Azeem, M. G. El Feky, M. Y. Hanfi

**Affiliations:** 1 Nuclear Power Plants Authority, 4 El Nasr Avenue, Nasr City, Cairo Egypt; 2 Faculty of Science, Al-Azhar University, P.O. Box 11884, Cairo, Egypt; 3 Nuclear Materials Authority, El Maadi, P.O. Box 530, Cairo, Egypt; 4 Ural Federal University, Mira Street 19, 620002 Ekaterinburg, Russia

**Keywords:** Environmental radioactive, Coastal plain, Black sand, Heavy economic minerals, Excess lifetime cancer risk, Principal component analysis (PCA), Ecology, Environmental sciences, Natural hazards, Signs and symptoms

## Abstract

This work is mainly concerned with the effect of anthropogenic activities and natural radioactivity due to the presence of highly radioactive black sand spots, factory construction, and shipping, in addition to other activities like agriculture on human beings. Forty samples were collected along Baltim–El Burullus coastal plain to detect the effect of these problems and determine the suggested solutions. The black sand of the Baltim–El Burullus coastal plain exhibits a considerable amount of economically heavy minerals, their ratio relative to the bulk composition in the investigated samples ranges from 3.18 to 10.5% with an average of 5.45%. The most important of them are magnetite, ilmenite, rutile, leucoxene, garnet, zircon and monazite. The existence of some radioactive-bearing accessory mineral deposits like zircon and monazite led to measuring the naturally occurring radionuclides ^226^Ra, ^232^Th and ^40^K to evaluate the excess lifetime cancer risk (ELCR). The results showed that these concentrations are 19.1 ± 9.73, 14.7 ± 9.53 and 211 ± 71.34 Bq kg^−1^ were lower than the corresponding reported worldwide average of 35, 45, and 412 Bq kg^−1^ for each radionuclide (^226^Ra, ^232^Th, and ^40^ K). The gamma hazard indices such as absorbed dose rate (D_air_), the annual effective dose (AED), and excess lifetime cancer risk (ELCR) factor were computed in the investigated sediments and all the results were found (D_air_ = 26.4 nGy h^−1^, AED = 0.03 mSv year^−1^, ELCR = 0.0001) to be lower than the values suggested by the United Nations Scientific Committee on the effect of Atomic Research (59 nGy h^−1^, 0.07 mSv year^−1^ and 0.0029 for D_air_, AED and ELCR, respectively). The study suggests that the black sand is safe to use in various infrastructure applications at Baltim–El Burullus coastal plain. The levels of radioactivity are not high enough to pose a risk to human health.

## Introduction

Naturally radioactive materials can be found in a variety of places in the environment, including rocks, soil, water, and air. Because natural radionuclides are a result of the Earth’s origin, there are no solutions to eliminate their presence^1,2^. To estimate the impacts of radiation exposure from both terrestrial and extraterrestrial sources, knowledge of radionuclide distribution and radiation levels in the environment is required. Despite the fact that these radionuclides are widely distributed, their concentrations are influenced by local geological conditions, which differ from one location to the next^3,4^.

These radionuclides expose people to radiation both outside and inside their homes. Gamma radiation causes external exposure from the ^238^U and ^232^Th series isotopes, as well as ^40^K, but inhalation of ^222^Rn, ^220^Rn, and their short-lived progeny, which produce alpha particles, causes internal exposure^5,6^. Radionuclide’s activity concentrations measurement in building materials is essential in assessing population exposure, as most individuals spend 80% of their time indoors^7,8^.

Mineral sand deposits in Egypt are found in various forms, beach deposits and coastal sand dunes. Sands with grades suitable for exploitation occur in the beach of Rosetta, Damietta, Baltim–El Burullus and north Sinai at El Arish^9–11^. The grade and reserves of the economic heavy minerals in the Egyptian black sands are appreciably high compared to other similar major world occurrences^12,13^. The mineralogy of the Egyptian black sands was studied previously by many workers.

The heavy mineral content of the El Burullus area was investigated by many authors^14,15^ recorded zircon, monazite, ilmenite, sphene, magnetite, cassiterite, garnet and monazite minerals, and concluded that El Burullus sediments appear to be slightly favourable delivery sites for thorium rather than uranium due to the enrichment of thorium—bearing minerals as monazite. El Alfi (2019) reported that the tonnage of total economic minerals in the West El Burullus area is about 850,500 tons, in which magnetite represents about 238,050 tons, ilmenite is about 477,900 tons, leucoxene is about 30,150 tons, rutile is about 18,000 ton, zircon with about 33,300 ton, monazite with about 2700 ton, titanite with about 2250 ton and garnet with about 48,150 ton. People residing in the study area use beach sand as building materials for their homes and other purposes. Therefore, it was necessary to study the effect of radionuclides on them^16,17^.

The present work aims to identify and study the physical properties of the heavy economic minerals and their distribution along the coastal plain of the Mediterranean Sea at Baltim–El Burullus coastal plain, and evaluate the radiological hazard indices, as well as the excess lifetime cancer risk (ELCR) resulting from the naturally occurring radionuclides ^226^Ra, ^232^Th and ^40^K that present in the black sand and hosted in the recorded economic minerals.

## Materials and methods

### Study area

**Figure 1:**
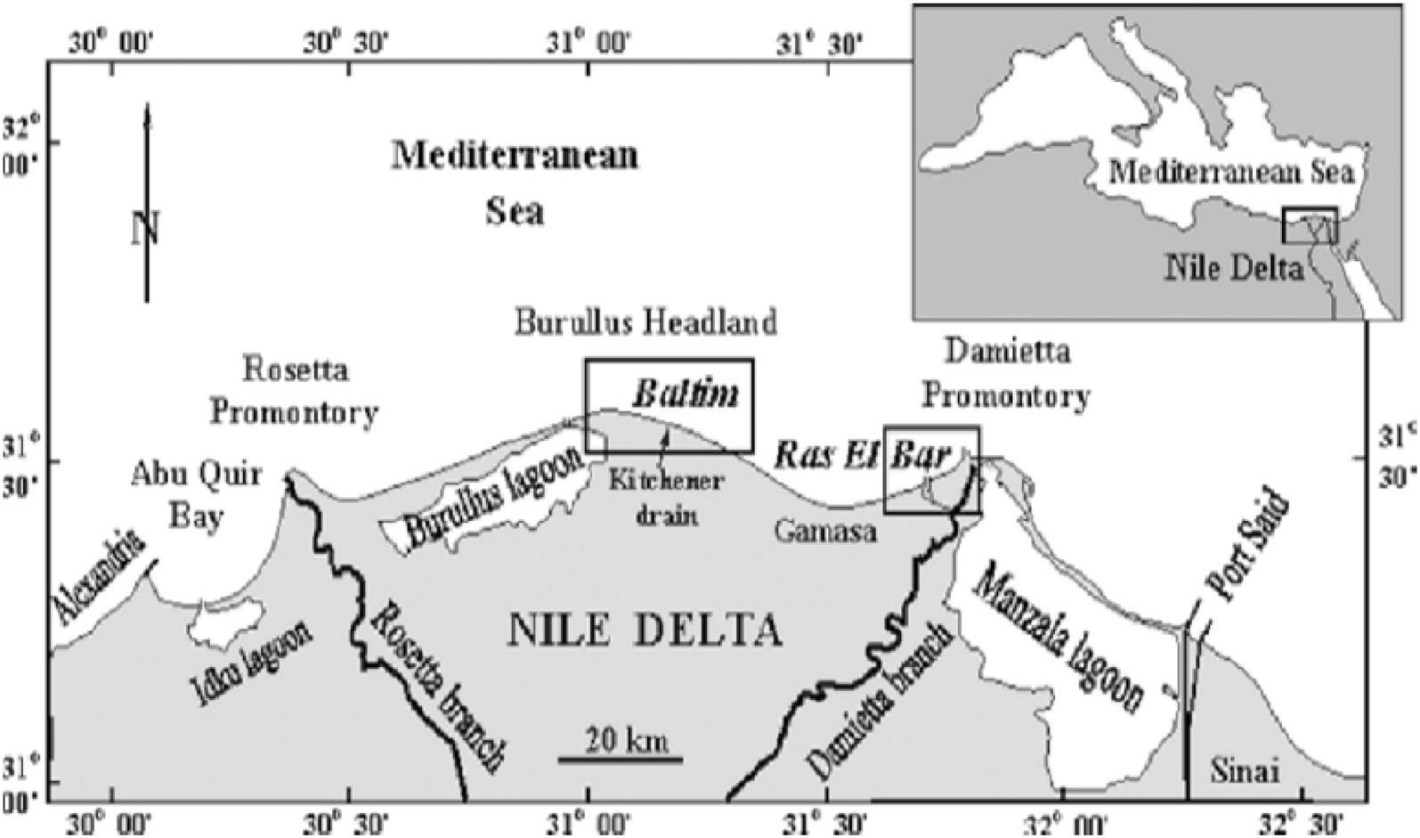
Landsat image of North Delta showing the study area.

The investigated area is located at the intersection of Long. 30° 00′ and 32° 30′ E and Lat. 31° 00′ and 32° 00′ N, in the coastal side of the north central Delta area (see Fig. 1), and had some geomorphological units such as the coastal plain, coastal sand dune, the beach, and cultivated lands. Its width ranges between 500 and 1500 m with very flat land in the area between Baltim and Gamsa. This plain is highly elevated in the Baltim area. The elevation of the foredune crest ranges between 10 and 35 m. a. s. l. On the stretch of beach between Baltim and El Burullus, forty samples were collected (Fig. 1), and nearly two to three samples were collected from each station perpendicular to the beach almost every 250 m according to the apparent changes in composition for accurate investigation of the studied area.

### Sampling and sample preparation

Four sampling patterns of the black sand, each reaching into the land for approximately m from the shoreline, were taken, with samples being spaced apart by about m. Along each profile, ten sediment samples were collected along the Baltim–El Burullus coastal plain (Fig. 1). Forty sediment samples were collected from four sampling profiles spaced in-between by about 50 m distance. Ten sediment samples were taken for each profile throughout the Baltim–El Burullus coastal plain (Fig. 1). The samples were physically gathered by turning a spiral rod to a depth of one meter, and each sample weighs between 5 and 7 kg. A GPS was used to determine the location of each sample. The samples were dispersed in four profiles along the Mediterranean coast from Baltim city to El Burullus. The distance between each sample is about five kilometers.

### Heavy minerals study

The heavy mineral constituents were separated from the investigated black sand using bromoform (specific gravity = 2.89 g cm^−3^). After separating different samples along Baltim–El Burullus coastal plain, glass funnels with filter papers were used for collecting the heavy fraction (sink layer), and the other one for collecting the light fraction (float layer). After complete filtration of the liquid, each fraction was washed with acetone to remove the residual bromoform in the interstices of the particles.

### Radioactivity measurements

To analyze samples are collected and transported to the lab in a plastic bag. Fine grains were formed by crushing the rock samples. A cylindrical plastic container with a diameter of 9.5 cm and height 3 cm was used to store the dried samples. After being sealed correctly, the container was left to settle in radioactive equilibrium for 28 days while containing free radon. Gamma-ray spectroscopy was used to calculate the percentages of U, Th, Ra, and K in the sediments under study. The NaI (Tl)-detector is characterized by high efficiency although it has a poor energy resolution. Its high efficiency allows fast and precise determinations of ^40^K, ^238^U, ^226^Ra and ^232^Th concentrations in rock and soil samples. The results depend on the accuracy of the energy calibration procedure that takes into account the possible interference of each nuclide in each peak region^18,19^. The gamma ray spectrometry system consists of a Bicron scintillation detector, NaI (Tl) crystal, 76 × 76 mm, hermetically sealed with a photomultiplier tube in aluminum housing.

The measurement of the radionuclides is based on choosing four energy regions of interest (ROIs) representing ^234^Th, ^212^Pb, ^214^Pb and ^40^K for U, Th, Ra and K, respectively. Uranium is estimated both as eU and Ra (eU) and thorium as eTh. The values of eU represent the concentration of U using the Th-234 energy peak (93 keV) and measure the first daughter isotope in the U238 decay series with very low possible loss. Radium is measured at the Pb-214 energy peak (352 keV) which is considered as a measure for c the concentration of the U only in case of the secular equilibrium state between U-238 and all its daughter isotopes. Thorium is measured at the Pb-212 energy peak (238 keV)^20^. The sediments samples were measured for 2000s using the minimum detection limits (MDL) of 2, 4, and 12 Bq kg^−1^ for ^238^U, ^232^Th, and ^40^K, respectively. The MDL for 238U, 232Th, and 40K are calculated for each sample by Eq. (1)^21^.

1 $$MDL = (2.7+ 4.65 \surd B )/( M\varepsilon I\gamma t),$$

where B is the count of the background below the peak of interest, ε is the absolute value efficiency, Iγ is the intensity of the gamma rays and t is counted. Time (seconds).

The radiological hazards variables are calculated according to Table 1.

**Table 1:** Important radiological parameters and indices^22,23^.

Parameter	Definition	Formula
D_air_ (nGy h^−1^)	The radioactive factor known as the absorbed dose rate was used to evaluate the effect of gamma radiation at a distance of 1 m from radiation sources in the air owing to the concentrations of ^238^U, ^232^Th, and ^40^K	D_air_ (nGy h^−1^) = 0.430 A_Ra_ + 0.666 A_Th_ + 0.042 A_K_
AED	An element of radioactivity called the yearly effective dose is used to gauge radiation exposure levels over a fixed period of time (1 year)	AED (mSv year^−1^) = D_air_ (nGy h^−1^) * 0.2 * 8760 (h year^−1^) * 0.7 (Sv Gy^−1^) * 10^–6^ (mSv nGy^−1^)
ELCR	The radioactive factor used to determine whether gamma radiation exposure caused lethal cancer is called excess lifetime cancer	ELCR = AED × DL × RF

## Results

### Mineralogical studies

#### Heavy and light fraction percentages

**Figure 2:**
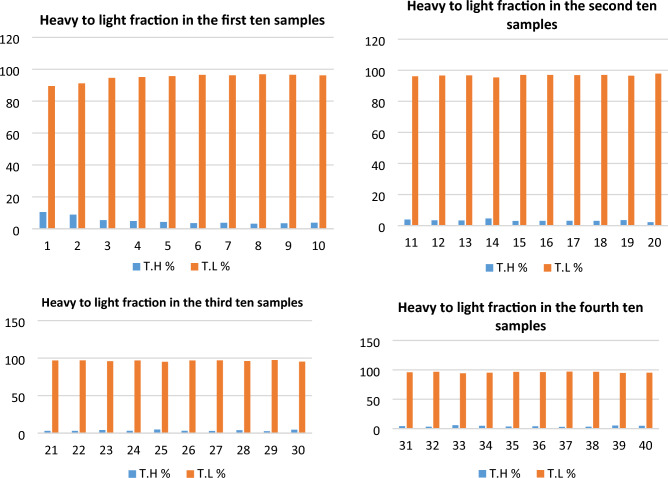
Total Heavy fraction (TH) and light fraction distribution along Baltim–El Burullus coastal plain.

After heavy liquid separation of the studied sediments, both heavy and light fractions were dried, weighted and their percentages were calculated and registered in Table 2 and Fig. 2.

**Table 2:** Distribution of total Heavy fraction (TH) and light fraction along Baltim–El Burullus coastal plain.

S. No.	T.H %	T.L %	S. No.	T.H %	T.L %	S. No.	T.H %	T.L %	S. No.	T.H %	T.L %
1	10.5	89.5	11	3.9	96.1	21	3.07	96.93	31	4.22	95.78
2	8.87	91.13	12	3.40	96.6	22	3.04	96.96	32	3.32	96.68
3	5.41	94.59	13	3.29	96.71	23	4.14	95.86	33	5.87	94.13
4	4.86	95.14	14	4.61	95.39	24	3.15	96.85	34	4.88	95.12
5	4.28	95.72	15	3.00	97	25	4.85	95.15	35	3.52	96.48
6	3.54	96.46	16	3.02	96.98	26	3.12	96.88	36	3.95	96.05
7	3.78	96.22	17	3.11	96.89	27	2.95	97.05	37	2.98	97.02
8	3.18	96.82	18	3.05	96.95	28	3.90	96.1	38	3.34	96.66
9	3.45	96.55	19	3.51	96.49	29	2.58	97.42	39	5.32	94.68
10	3.83	96.17	20	2.15	97.85	30	4.61	95.39	40	4.85	95.15
Min	3.18	89.5	Min	2.15	95.39	Min	2.58	95.15	Min	2.98	94.13
Max	10.5	96.82	Max	4.61	97.85	Max	4.85	97.42	Max	5.87	97.02
Av	5.45	94.55	Av	3.32	96.68	Av	3.57	96.43	Av	4.26	95.74

Using microscopic analysis and an ESEM connected with an EDX micro-analyzer, the mineralogy and chemistry of the segregated mineral grains were identified.

### Mineral analysis

**Figure 3:**
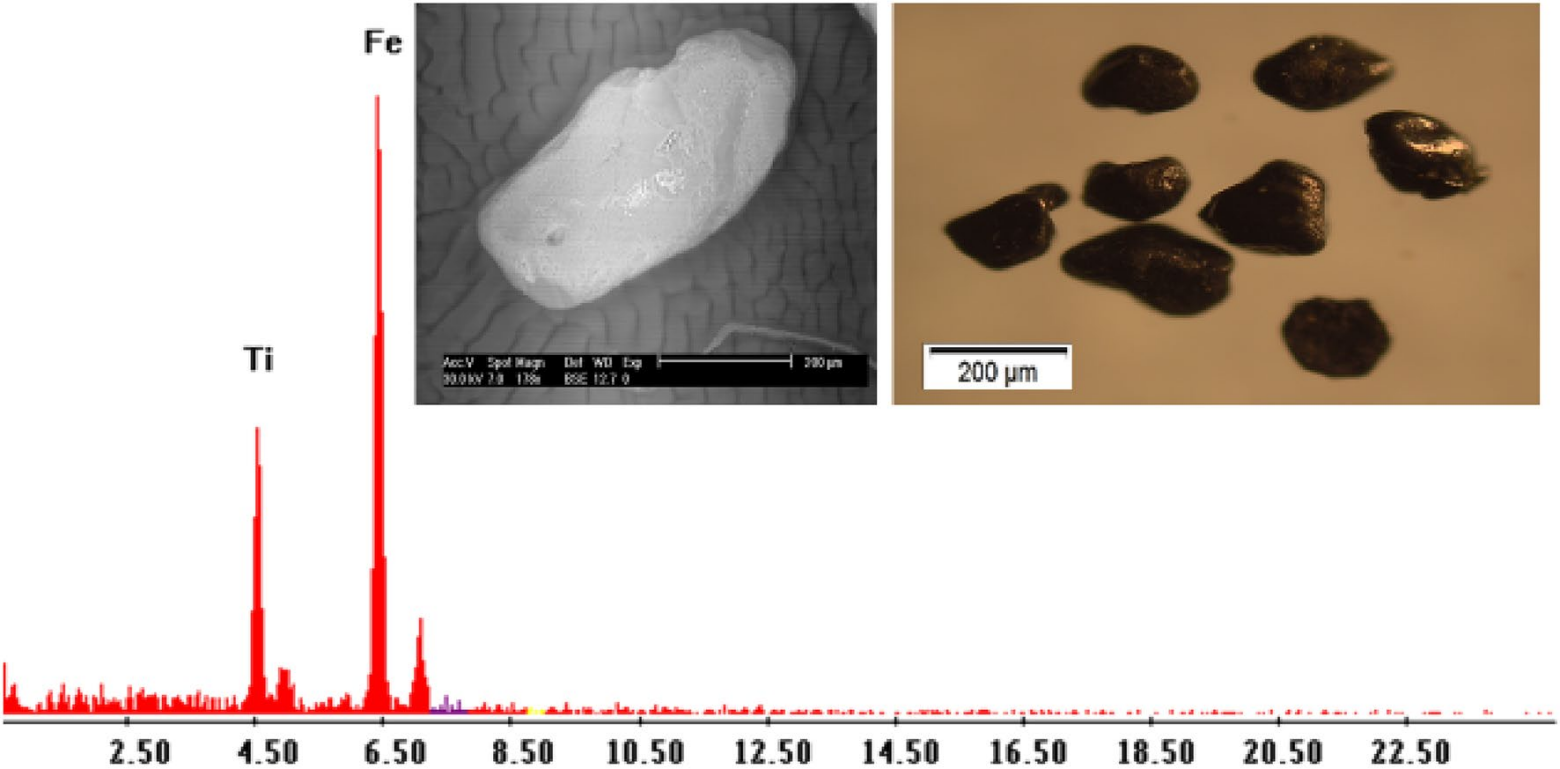
BSE image, EDX pattern and photomicrograph of ilmenite.

**Figure 4:**
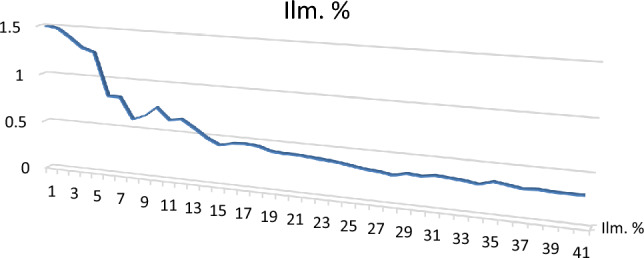
Distribution of ilmenite in the studied stream sediments.

The following mineral phases were identified from the heavy fraction using Binuclear and Environmental Scan Electron Microscope techniques:

**Ilmenite (FeTiO**_**3**_**)** is present as an accessory mineral associated with hematite and magnetite. Ilimenite grains are subhedral iron black or asphaltic in color opaque grains, with a bluish or violet tint. It is the most abundant mineral in the studied sediments. The percentages of Ilmenite minerals in the studied sediments ranged from 0.3 to 1.5% with an average of 0.57% (Table 3; Figs. 3, 4). The EDX data show that it is essentially made up of Ti (62.15%) and Fe (35.35%) with small amounts of Si and Ca.

**Table 3:** Heavy minerals distribution along Baltim–El Burullus coastal plain.

S. No.	Mag. %	Ilm. %	Rut. %	Leuc. %	Gar. %	Zr. %	Mz. %
1	0.5	1.5	0.3	0.25	0.25	0.04	0.03
2	0.4	1.48	0.2	0.22	0.23	0.033	0.025
3	0.4	1.4	0.23	0.23	0.21	0.032	0.023
4	0.3	1.3	0.18	0.21	0.19	0.031	0.021
5	0.3	1.26	0.16	0.20	0.2	0.03	0.019
6	0.25	0.82	0.17	0.21	0.17	0.024	0.014
7	0.23	0.82	0.19	0.20	0.14	0.022	0.012
8	0.21	0.60	0.16	0.19	0.145	0.021	0.011
9	0.20	0.65	0.15	0.17	0.13	0.021	0.01
10	0.20	0.75	0.16	0.14	0.12	0.020	0.01
11	0.21	0.63	0.13	0.12	0.12	0.017	0.01
12	0.20	0.65	0.15	0.11	0.10	0.015	0.01
13	0.14	0.57	0.12	0.10	0.12	0.013	0.01
14	0.12	0.48	0.11	0.10	0.11	0.011	0.01
15	0.11	0.42	0.07	0.10	0.07	0.011	0.01
16	0.10	0.45	0.05	0.10	0.065	0.011	0.01
17	0.11	0.46	0.08	0.11	0.08	0.01	0.007
18	0.11	0.45	0.06	0.10	0.06	0.01	0.005
19	0.09	0.41	0.07	0.10	0.07	0.01	0.003
20	0.10	0.40	0.05	0.08	0.05	0.01	0.0022
21	0.10	0.40	0.04	0.075	0.04	0.01	0.002
22	0.08	0.39	0.04	0.05	0.04	0.01	0.001
23	0.10	0.38	0.03	0.06	0.03	0.001	0.001
24	0.10	0.37	0.02	0.06	0.02	0.001	0.001
25	0.10	0.35	0.04	0.04	0.021	0.0015	0.0011
26	0.10	0.33	0.03	0.05	0.02	0.0012	0.001
27	0.10	0.32	0.01	0.05	0.01	0.001	0.001
28	0.05	0.30	0.04	0.04	0.01	0.001	0.0012
29	0.03	0.33	0.02	0.04	0.01	0.001	0.0011
30	0.04	0.32	0.01	0.01	0.01	0.01	0.001
31	0.02	0.34	0.01	0.012	0.01	0.001	0.001
32	0.01	0.33	0.04	0.013	0.01	0.0012	0.0012
33	0.04	0.32	0.03	0.01	0.012	0.0014	0.0013
34	0.04	0.30	0.03	0.01	0.013	0.0011	0.001
35	0.02	0.34	0.04	0.015	0.01	0.0015	0.0012
36	0.03	0.32	0.02	0.01	0.011	0.0012	0.0011
37	0.07	0.30	0.04	0.01	0.01	0.0012	0.0012
38	0.04	0.31	0.04	0.016	0.01	0.0012	0.0011
39	0.06	0.30	0.04	0.013	0.01	0.001	0.001
40	0.05	0.30	0.03	0.02	0.01	0.0011	0.001
Min	0.010	0.300	0.010	0.010	0.010	0.001	0.001
Max	0.500	1.500	0.300	0.250	0.250	0.040	0.030
Av	0.142	0.570	0.088	0.093	0.076	0.011	0.007

**Figure 5:**
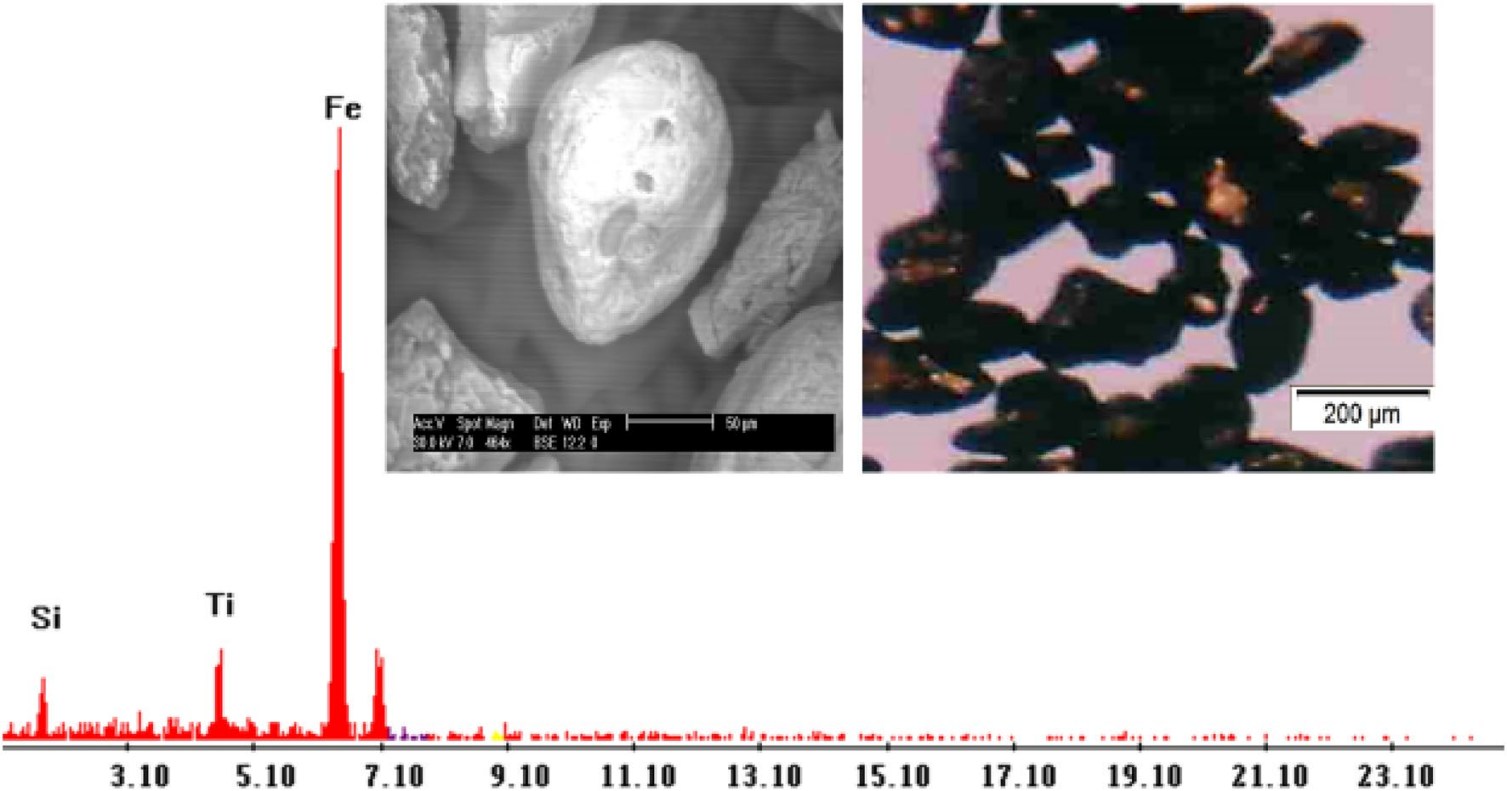
BSE image, EDX pattern and microphotograph of magnetite.

**Figure 6:**
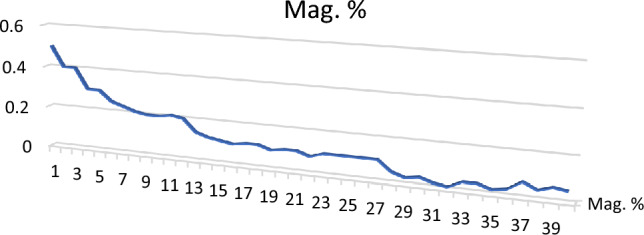
Distribution of magnetite in the studied stream sediments.

Magnetite (Fe^2+^ Fe^3+^_2_O_4_): It is obtained by a small hand magnet and its percentage varies from 0.01 to 0.5% with 0.14 as an average (Table 3; Figs. 5, 6). It is the second constituent in abundance after ilmenite and occurs as black color grains with rounded to subrounded edges. It is mainly composed of Fe (85%) with a minor amount of Ti and Si.

**Figure 7:**
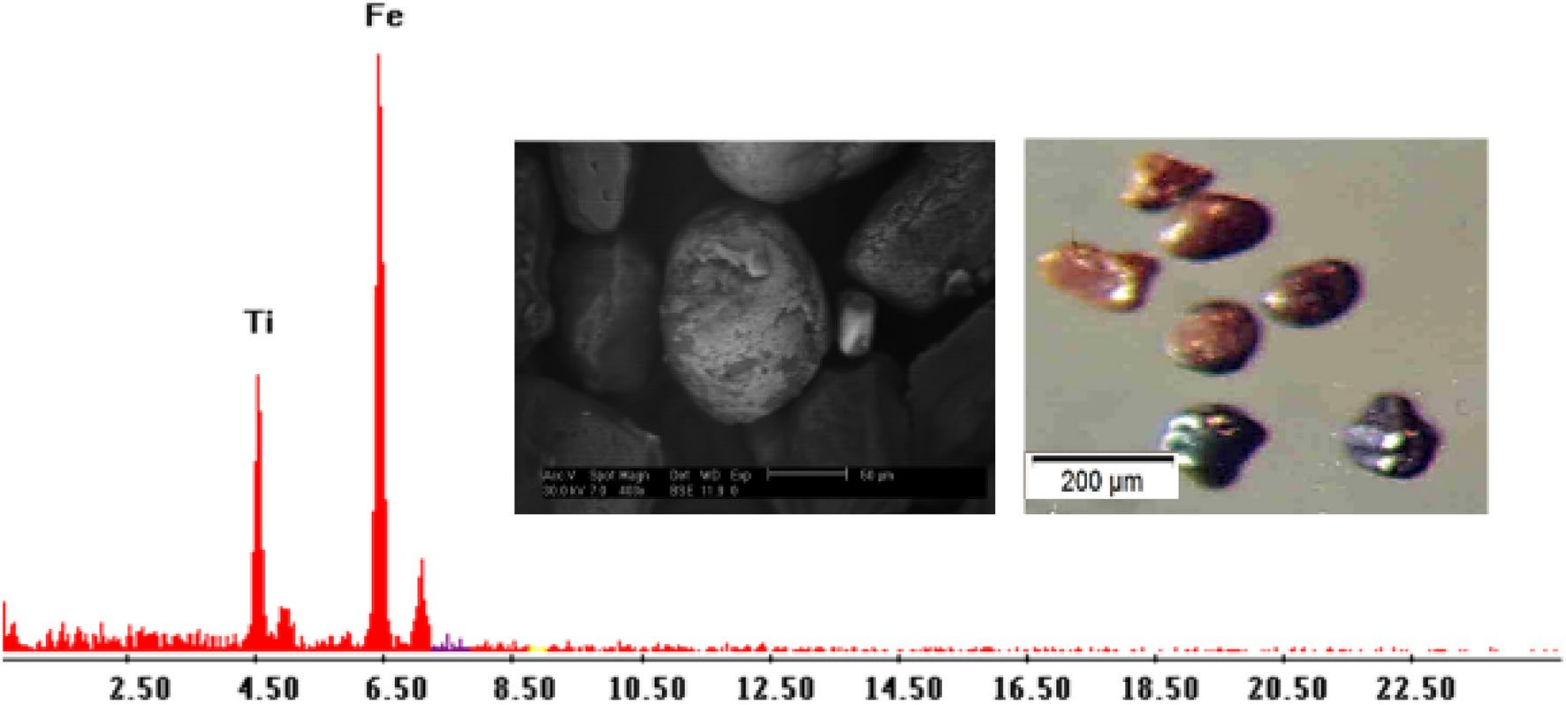
BSE image, EDX pattern and photomicrograph of leucoxene.

**Figure 8:**
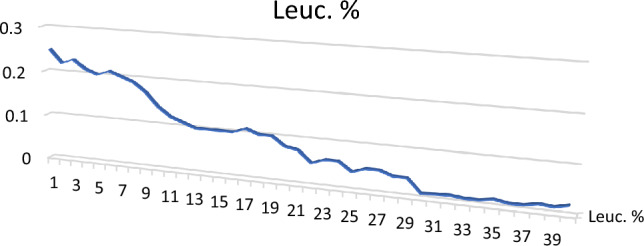
Distribution of leucoxene in the studied stream sediments.

Leucoxene: Ilmenite undergoes modification to produce leucoxene, which is the intermediate step between ilmenite and secondary rutile and often appears as spherical grains that are opaque to transmitted light. Leucoxene percentages in the studied sediments ranged from 0.01 to 0.25% with an average of 0.093% (Table 3; Figs. 7, 8). Colors of leucoxene range from yellowish brown to white. Most of the leucoxene grains have smooth waxy surfaces, however, some of them show pitted surfaces and light creamy color with Ti-rich content The EDX data show the composition of leucoxene with Ti (43.64%), Fe (53.89%).

**Figure 9:**
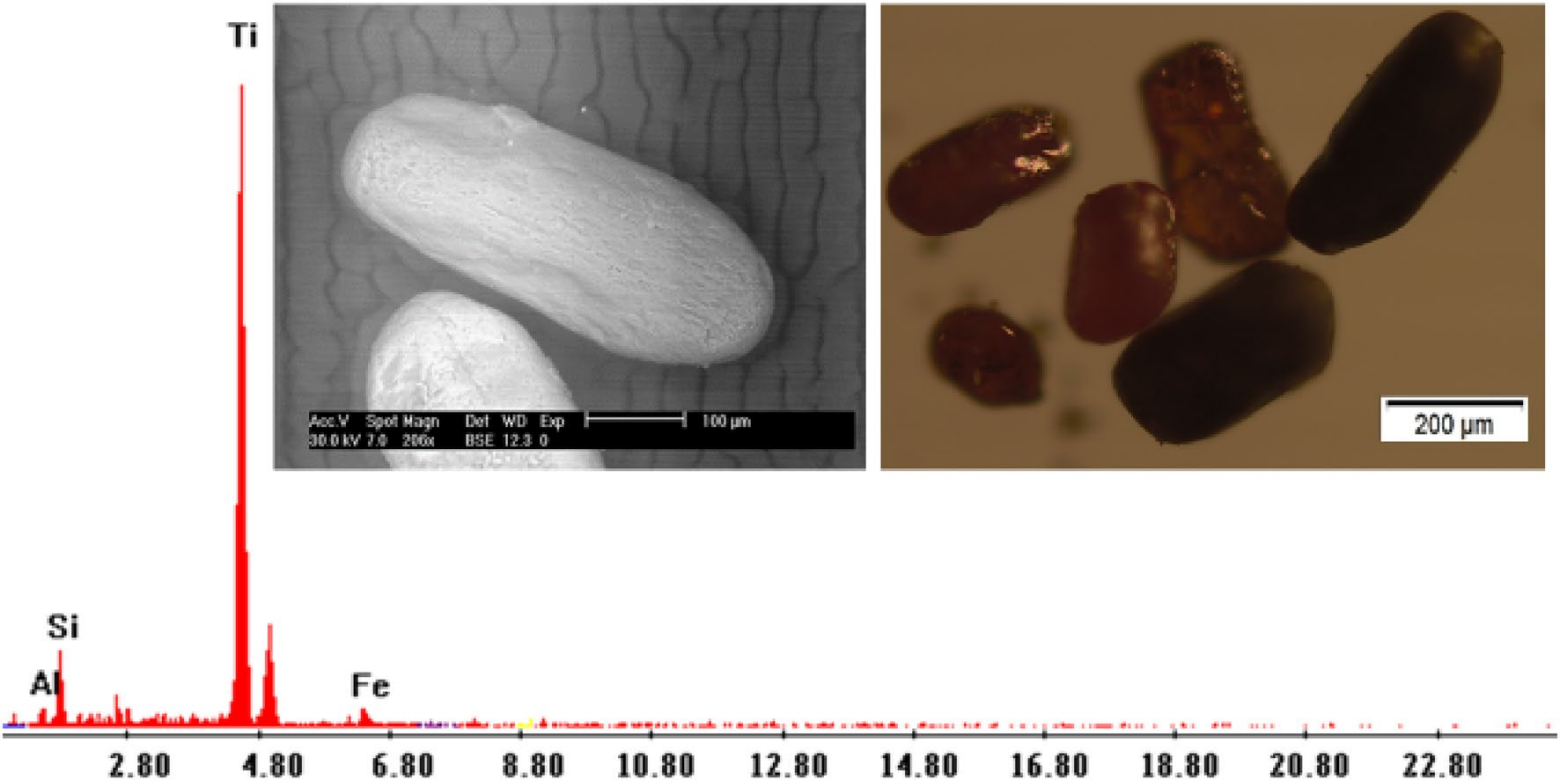
BSE image, EDX pattern and photomicrograph of rutile.

**Figure 10:**
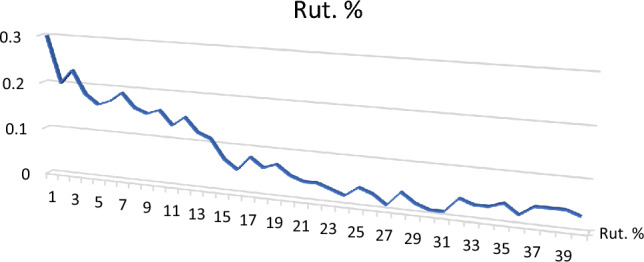
Distribution of rutile in the studied stream sediments.

Rutile (TiO_2_): It occurs as elongated and prismatic crystals concentrated in the non-magnetic and magnetic fractions at 1.5 amp. It has a black color to reddish brown to opaque color. Its average percentage in the studied sediments reaches up to 0.088% (Table 3; Figs. 9, 10).

It exists as subhedral, subrounded reddish black grains having Ti (85.17%) as the main composition, with small amounts of Fe, Si and Al. This variation in habits indicates that the recorded rutile grains are inherited from different sources.

**Figure 11:**
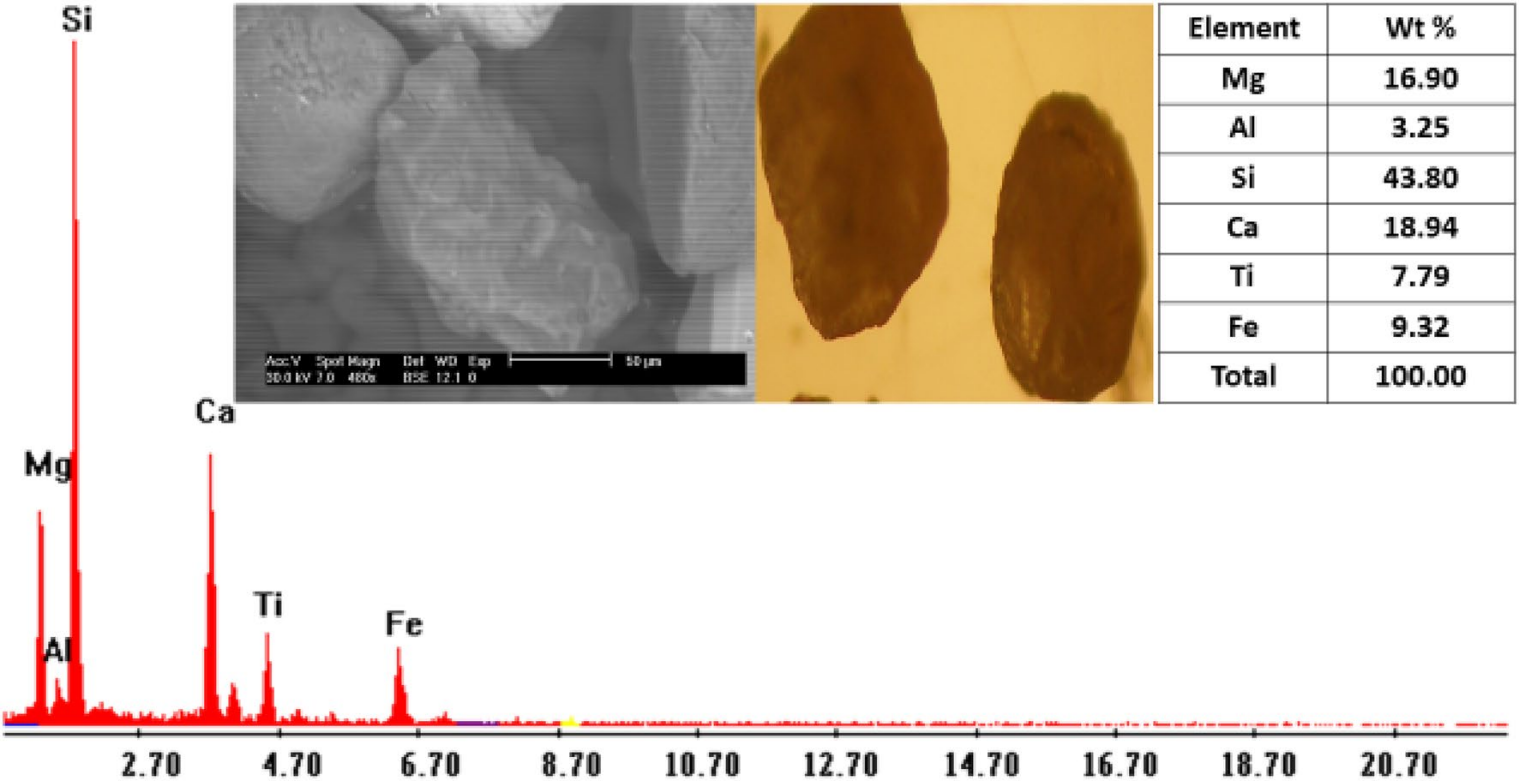
BSE image, EDX pattern and photomicrograph of garnet.

**Figure 12:**
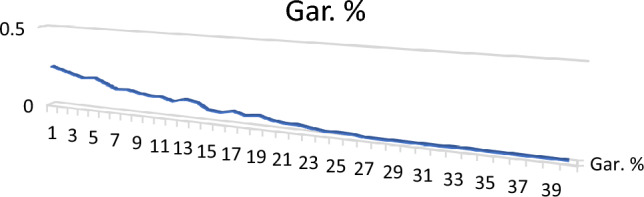
Distribution of garnet in the studied stream sediments.

Garnet (Fe_3_Al _2_Si_3_O_12_): Garnet of stream sediments in the studied area are mainly of alamandine[Fe_3_A1_2_(SiO_4_)] type. It is a group of isomorphous minerals with different compositions and colors, and have nearly identical physical and chemical properties. It was found as colourless, brand wn, rose, red, and subrounded to well-rounded grains. The percentages of garnet minerals in the studied black sands are ranging from 0.01 to 0.25% with an average of 0.08% (Table 3; Figs. 11, 12). EDX analyses clarify that the std garnets mainly contain Si, Ca, Mg, Fe, Ti and Al.

**Figure 13:**
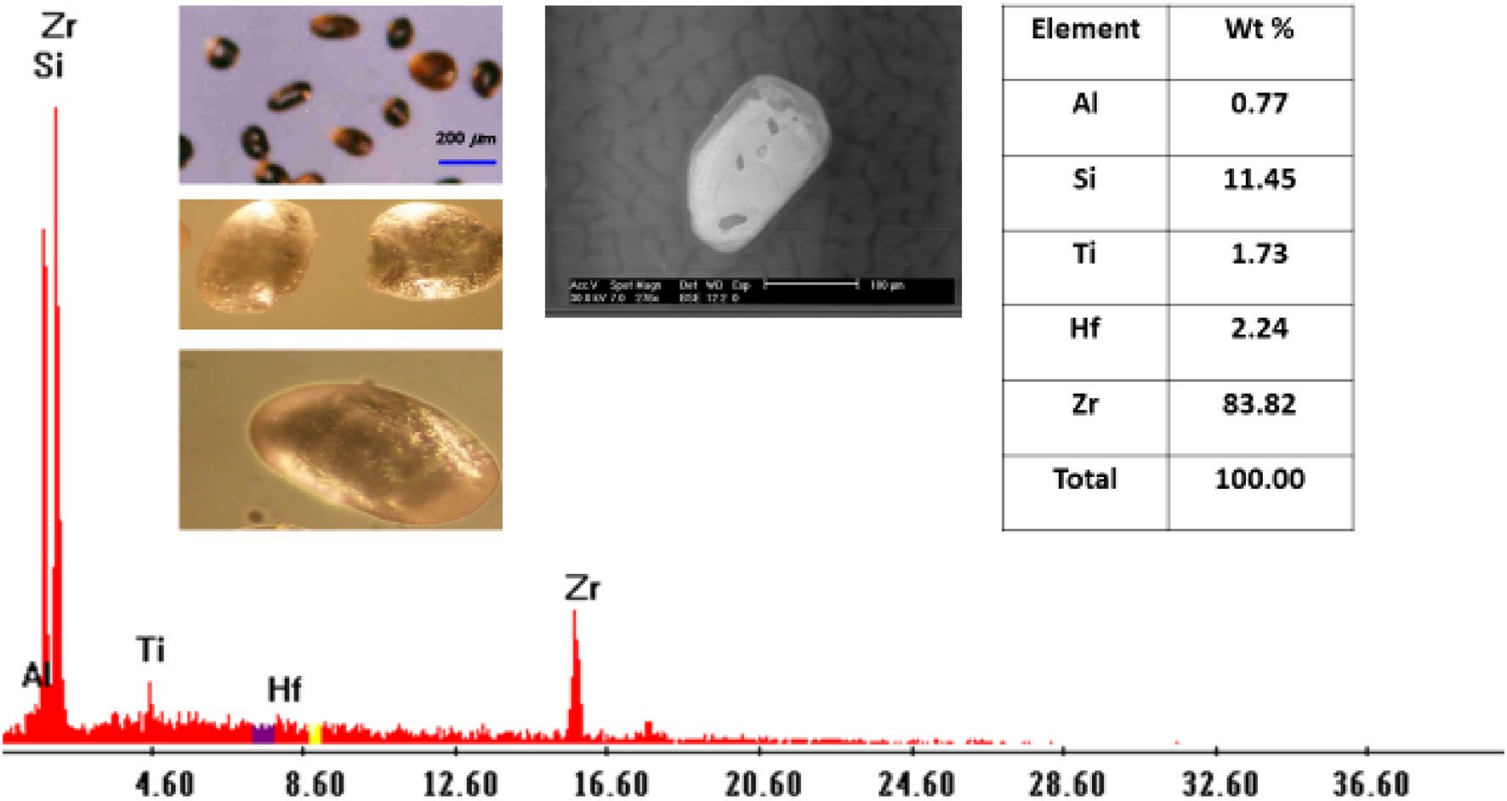
BSE image, EDX pattern and photomicrograph of zircon.

Zircon: (ZrSiO_4_) in the studied sediments occurs frequently, either as short or long prismatic ultra-stable crystals with or without bipyramidal terminations and has various colors (pale yellow, reddish brown, reddish-orange and colorless). Uranium may be the most important trace element in zircon^24,25^. The presence of high contents of U in zircon leads to the breakdown of the structure of zircon (metamict state), which causes radial and concentric fractures that are good pathways for uranium addition in presence of iron oxy-hydroxides^24,26^. Dardier^27^ stated that zircon could be used as a guide for U mineralization. The EDX analyses for these crystals reflect the chemical composition of zircon. It is mainly composed of Zr (83.82%), Hf (2.24%), Si (11.45%) with appreciable amounts of Al and Ti (Table 3; Figs. 13, 15).

**Figure 14:**
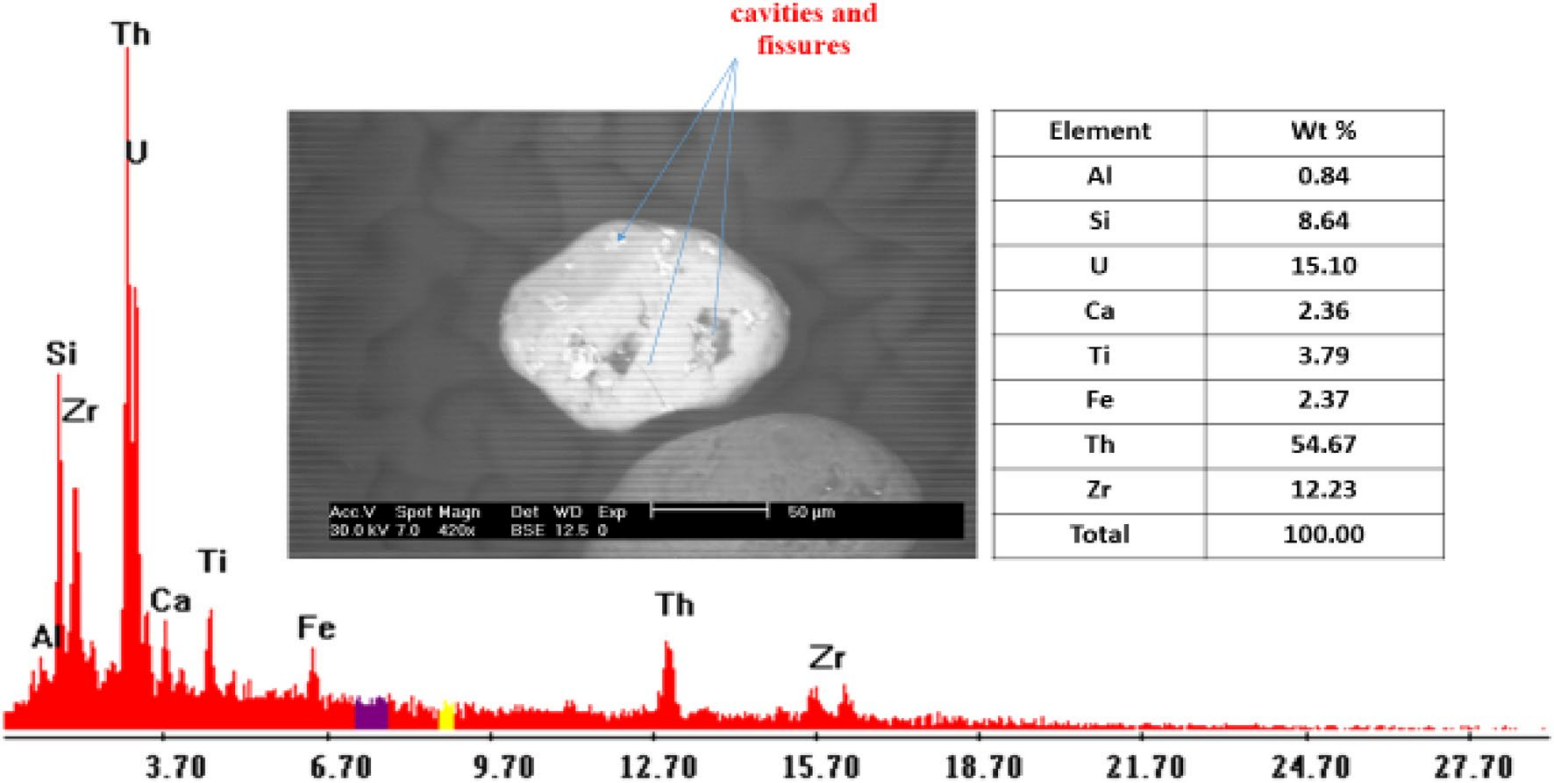
BSE image and EDX pattern of uranothorite.

**Figure 15:**
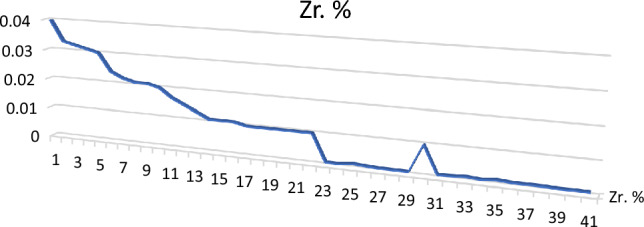
Distribution of zircon in the studied stream sediments.

Zircon–Thorite intergrowth: The most metamictization-prone minerals are zircon and thorite because of their radio-element concentrations. To form zirconian thorite, thorian zircon, yttrian zircon, and yttrian thorite, solid solutions with distinct ranges could therefore develop from these two minerals. When metamictized overgrowth zircon crystals completely dissolve, it is sometimes noted because zircon fragments are still present in the crystals^28–31^. Fe-dominant oxide/hydroxide phases, which have a goethite-like composition, partially or entirely fill cavities and fissures in the zircon under study. Admixtures of Si, Al, Mg, Ti, and Ca are also visible (Fig. 14). The EDX analysis indicates that zirconium uranothorite crystals are essentially composed of Th (54.67%), U (15.10%), Zr (12.23%), in addition to Ca, Ti, Fe, and Al (Fig. 15).

**Figure 16:**
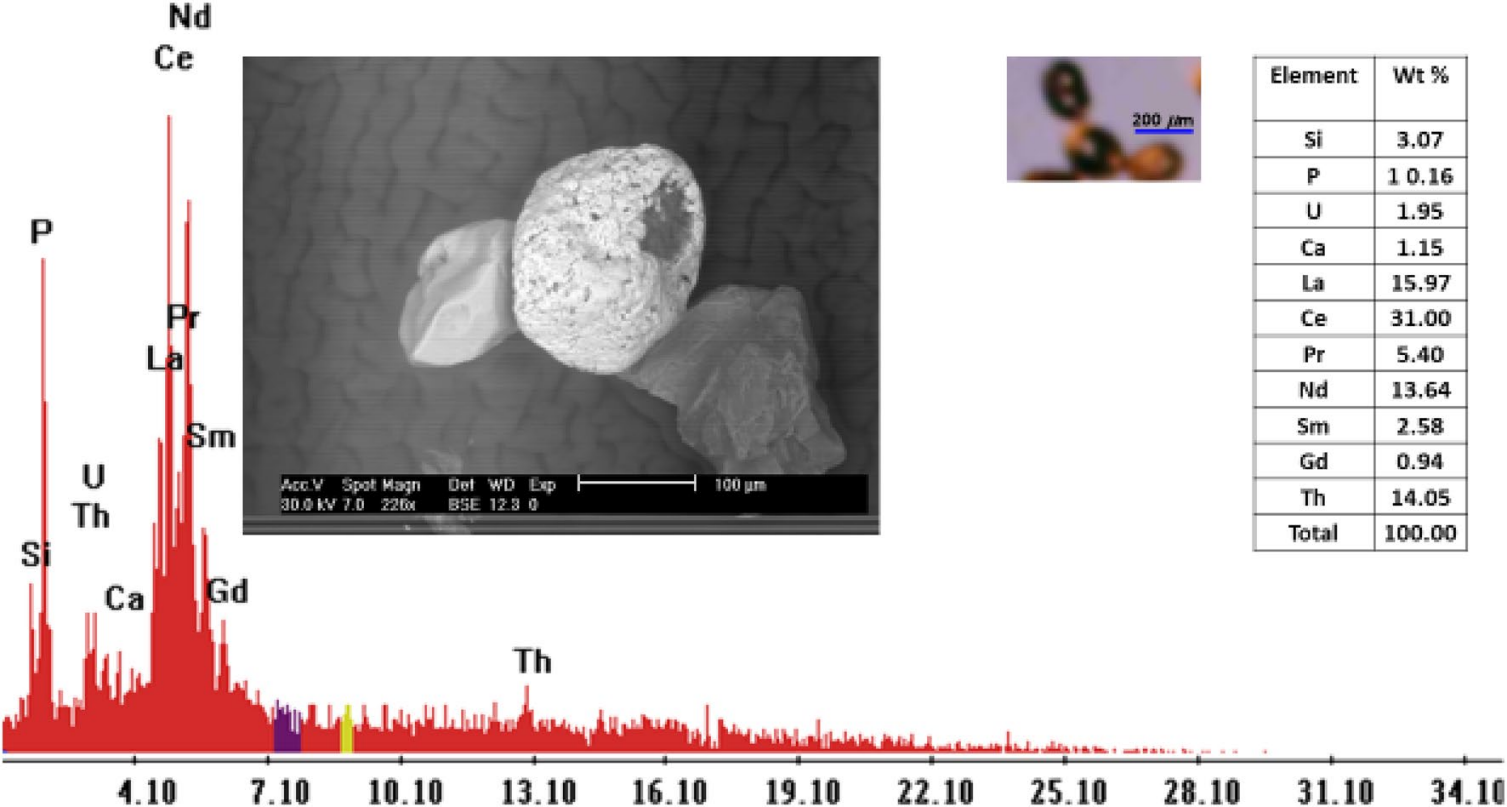
BSE image, EDX pattern and photomicrograph of monazite.

**Figure 17:**
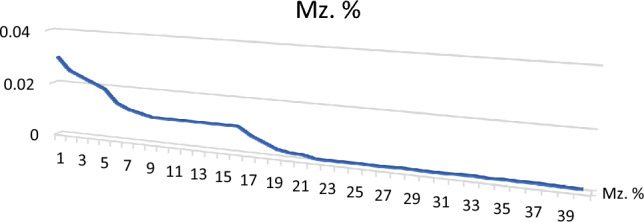
Distribution of monazite in the studied stream sediments.

Monazite {(Ce, La, Nd, Th)PO_4_}: Occur in the fine sand size as rounded grains, translucent of honey yellow color or colorless. Monazite is a phosphate mineral which attains special importance because of its rare metal contents like thorium and REEs, especially cerium and lanthanum while uranium is present in small amounts^32^. It is frequently present in different igneous and metamorphic rocks^33^. Ce-monazite is abundant in the studied stream sediments. The monazite crystals are found in small amounts and their reaches reach up to 0.03%. EDX analyses indicate that monazite elemental composition includes Ce, La, Nd, Th, Sm, U, Ca, P and Si (Figs. 16, 17; Table 3).

**Figure 18:**
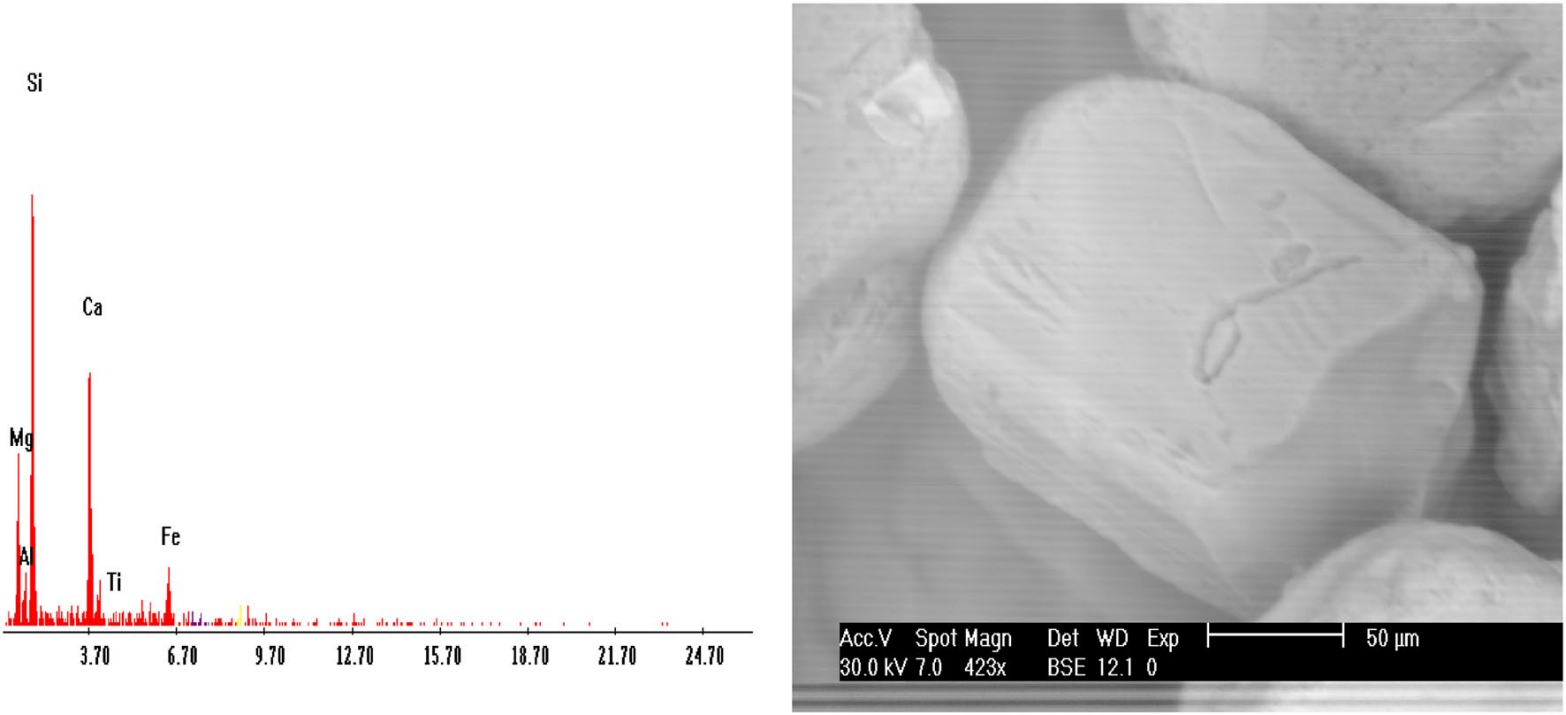
BSE image and EDX pattern of pyroxene.

Other heavy minerals Pyroxene XY(Si, Al)_2_O_6_: It is found in the study area in which Mg represents 15.58%, Ca (25.54%), Fe (9.35%), Si (43.44%), Al (5%) with few amount of Ti. Also, apatite, cassiterite, titanite (Sphene), pyrite and hematite were recorded in the studied sediments (Fig. 18).

### Radioactivity study

It is crucial to understand the level of natural radioactivity in these sediments. In order to assess the radiological hazards indices (RHI) and excess lifetime cancer risk, concentrations of naturally occurring radionuclides (^226^Ra, ^232^Th, and ^40^K) in the sediments of the Baltim–El Burullus coastal plain were measured (ELCR) (Table 4). As revealed in Table 4, the mean data of ^226^Ra, ^232^Th and ^40^K activity concentrations are 19.1 ± 9.73, 14.7 ± 9.53 and 211 ± 71.34 Bq kg^−1^, respectively, which it is lower than the recommended worldwide value 33, 45 Bq kg^−1^, while ^40^K activity concentrations are higher than recommended worldwide limit 412^25,34^. The values of ^226^Ra activity concentrations altered between 11.1 and 44.4 Bq kg^−1^. The Min and Max values of ^232^Th are 4 and 40.4 Bq kg^−1^, respectively. Moreover, the variation of ^40^K values altered from 65.7 to 391.3 Bq kg^−1^. The highest values of activity concentrations of ^226^Ra, ^232^Th and ^40^K were recorded in the investigated black sand due to the high radioactivity of altered sand is referred to the occurrence of zircon, monazite, magnetite, ilmenite, rutile, leucoxene, and garnet.

**Table 4:** Activity concentrations (Bq kg^−1^) of ^226^Ra, ^232^Th and ^40^K of the investigated black sand samples.

Sample code No.	Activity concentration (Bq kg^−1^)			D_air_ (nGy h^−1^)	AED (mSv year^−1^)	ELCR × 10^–3^
	^226^Ra	^232^Th	^40^K			
1	11.1 ± 3.1	8.08 ± 2.8	237.88 ± 88.6	19.8	0.02	0.1
2	11.1 ± 3.1	12.12 ± 4.3	147.11 ± 33.5	18.5	0.02	0.1
3	11.1 ± 3.1	12.12 ± 4.3	147.11 ± 33.5	18.5	0.02	0.1
4	33.3 ± 12.6	4.04 ± 0.9	200.32 ± 41.3	26.0	0.03	0.1
5	33.3 ± 12.7	24.24 ± 9.4	222.23 ± 74.3	39.1	0.05	0.2
6	33.3 ± 12.8	12.12 ± 4.3	147.11 ± 33.5	28.7	0.04	0.1
7	22.2 ± 7.4	32.32 ± 11.3	200.32 ± 41.3	38.0	0.05	0.2
8	22.2 ± 7.4	24.24 ± 9.4	284.83 ± 103.4	36.6	0.04	0.2
9	11.1 ± 3.1	8.08 ± 2.8	181.54 ± 56.6	17.5	0.02	0.1
10	22.2 ± 7.4	12.12 ± 4.3	275.44 ± 94.3	28.9	0.04	0.1
11	11.1 ± 3.1	16.16 ± 5.2	84.51 ± 23.4	18.4	0.02	0.1
12	11.1 ± 3.1	16.16 ± 5.2	259.79 ± 91.2	25.5	0.03	0.1
13	22.2 ± 7.4	8.08 ± 2.8	228.49 ± 56.7	24.5	0.03	0.1
14	11.1 ± 3.1	12.12 ± 4.3	391.25 ± 124.3	28.5	0.03	0.1
15	22.2 ± 7.4	12.12 ± 4.3	262.92 ± 92.3	28.4	0.03	0.1
16	22.2 ± 7.4	12.12 ± 4.3	197.19 ± 66.4	25.7	0.03	0.1
17	33.3 ± 12.7	8.08 ± 2.8	122.07 ± 26.5	25.3	0.03	0.1
18	22.2 ± 7.4	12.12 ± 4.3	200.32 ± 41.3	25.8	0.03	0.1
19	44.4 ± 19.3	40.4 ± 17.4	228.49 ± 75.4	54.3	0.07	0.2
20	22.2 ± 7.4	8.08 ± 2.8	184.67 ± 58.2	22.7	0.03	0.1
21	44.4 ± 19.3	8.08 ± 2.8	150.24 ± 43.7	31.6	0.04	0.1
22	33.3 ± 12.7	36.36 ± 14.2	184.67 ± 58.2	44.9	0.06	0.2
23	11.1 ± 3.1	16.16 ± 5.2	250.4 ± 89.2	25.2	0.03	0.1
24	11.1 ± 3.1	16.16 ± 5.2	241.01 ± 78.9	24.8	0.03	0.1
25	11.1 ± 3.1	4.04 ± 0.9	122.07 ± 26.5	12.6	0.02	0.1
26	11.1 ± 3.1	4.04 ± 0.9	278.57 ± 99.4	19.0	0.02	0.1
27	22.2 ± 7.4	24.24 ± 9.4	203.45 ± 42.3	33.2	0.04	0.1
28	11.1 ± 3.1	12.12 ± 4.3	165.89 ± 46.5	19.3	0.02	0.1
29	11.1 ± 3.1	4.04 ± 0.9	325.52 ± 112.3	20.9	0.03	0.1
30	22.2 ± 7.4	8.08 ± 2.8	253.53 ± 86.4	25.5	0.03	0.1
31	11.1 ± 3.1	8.08 ± 2.8	253.53 ± 86.4	20.4	0.03	0.1
32	22.2 ± 7.4	4.04 ± 0.9	294.22 ± 89.5	24.8	0.03	0.1
33	11.1 ± 3.1	24.24 ± 9.4	266.05 ± 90.2	30.7	0.04	0.1
34	11.1 ± 3.1	12.12 ± 4.3	159.63 ± 53.4	19.0	0.02	0.1
35	11.1 ± 3.1	16.16 ± 5.2	128.33 ± 27.4	20.2	0.02	0.1
36	11.1 ± 3.1	8.08 ± 2.8	65.73 ± 21.2	12.7	0.02	0.1
37	22.2 ± 7.4	12.12 ± 4.3	165.89 ± 46.5	24.4	0.03	0.1
38	22.2 ± 7.4	20.2 ± 6.2	150.24 ± 43.7	28.6	0.04	0.1
39	11.1 ± 3.1	16.16 ± 5.2	200.32 ± 41.3	23.1	0.03	0.1
40	11.1 ± 3.1	40.4 ± 17.4	375.6 ± 180.4	44.9	0.06	0.2
Mean	19.1	14.7	211.0	26.4	0.03	0.1
SD	9.73	9.53	71.34	8.74	0.01	0.04
Min	11.1	4.0	65.7	12.6	0.02	0.05
Max	44.4	40.4	391.3	54.3	0.1	0.2
Egypt, Lake Nasser*	21.0	23.0	155.0	N. M	N. M	N. M
Nile Delta and Middle Egypt**	18.0	17.0	316.0	N. M	N. M	N. M
Nile River sediments from Aswan to Minya***	16.3	12.9	200.2	N. M	N. M	N. M
World average in sediments****	33.0	45.0	420.0	N. M	N. M	N. M

*Data after^35^. **Data after^36^. ***Data after^37^. ****Data after^38^. *N.M* not measures.

When average concentration values compared to those of different sediments of Nile Valley the Baltim El Burullus coastal plain sediments are higher average concentration of ^226^Ra, ^232^Th than the Nile Delta, Middle Egypt and sediments from Aswan to Minya and lower than those of Lake Nasser, and vice versa for the average concentration of ^40^K (Table 4).

The D_air_ in the studied sediments ranges from 12.6 to 54.3 nGy h^−1^ with an average of 26.4 nGy h^−1^, which less than the population weighted average 59 nGy h^−1^^38^ and annual effective dose, AED ranges from 0.02 to 0.1 mSv year^−1^ with an average of 0.03 mSv year^−1^, which are less than the acceptable value of (0.07 mSv year^−1^) for the outdoor annual effective dose by UNSCEAR^38^.

There is some evidence that long-term radiation exposure increases the chance of developing cancer^39^. A person may be at an increased risk of developing cancer if they are exposed to cancer-causing substances for a longer period of time (ELCR). The ELCR factor evaluated in the current study spans from 0.05 × 10^–3^ to 0.2 × 10^–3^ with an average of 0.1 × 10^–3^, which is lower than the global average of 2.9 × 10^–3^ (quoted by^39,40^).

## Discussion

### Mineral investigation

The black sand of the Baltim–El Burullus coastal plain exhibits a considerable amount of economic heavy minerals, their ratio relative to the bulk composition in the investigated samples ranges from 3.18 to 10.5% with an average of 5.45%. The most important of them are magnetite, ilmenite, rutile, leucoxene, garnet, zircon and monazite. Also, pyroxene, apatite, cassiterite, titanite, pyrite and hematite minerals present with minor concentrations.

The most predominant mineral in the studied samples is ilmenite which ranges from 0.3 to 1.5% with an average of 0.570%, the second predominant mineral is magnetite which ranges from 0.01 to 0.5% with an average of 0.142%, and the percentage of each mineral in the rest of economic minerals does not exceed 0.1%. Leucoxene ranges from 0.010 and 0.3% with an average of 0.093%, rutile ranges from 0.01 to 0.3 with an average of 0.088, and garnet ranges from 0.01 to 0.25% with an average of 0.076. The least predominant minerals are zircon and monazite, respectively. Zircon ranges from 0.001 and 0.40% with an average of 0.011%, while monazite ranges from 0.001 to 0.30% with an average of 0.007%.

The mineralogical investigation using the ESEM shows that the recorded minerals have different habits (Euhedral to rounded) indicating that they were inherited from different sources, garnet minerals are generally used in characterizing various metamorphic facies. The rounded habit for magnetite, leucoxene, garnet and monazite indicates the long distance of transportation. The zircon euhedral shape clarifies its magmatic origin. Armstrong (1922), Groves (1930), Poldervart (1955–1956), Saxena (1966) and Zircon crystals are mild to moderately rounded at one or both ends, according to Ebyan et al.^41^, and dismissals of the pyramidal and/or primal faces may be connected to late magmatic corrosion or late hydrothermal action. Some zircon grains are metamict that indicates the presence of high uranium content in their structure, other metamict zircon crystals are completely transformed into zirconian thorite by acidified solutions rich in Th, which react with the original zircon crystals through cavities and fissures^41^. In addition, the monazite mineral in the investigated sediments attains special importance because of its thorium. River sediments are a substantial source of radioactivity that greatly raises the background radiation level^40^.

### Radioactive analysis

As can see in Table 5, the mean activity concentrations of the ^226^Ra, ^232^Th, and ^40^K radionuclides are lower than the recommended worldwide average^21,42^. This pushes us to perform the statistical analysis (Table 5). According to the statistical investigation, the Skewness, kurtosis and coefficient variance for the obtained results of radionuclides activity concentrations are presented. Table 5 illustrate that the skewness of the ^226^Ra, ^232^Th, and ^40^K activity concentrations are positive values. Therefore, the distributions of ^226^Ra, ^232^Th, and ^40^K are asymmetric in nature. In addition to the kurtosis values indicate the peakness of a probability distribution. According to Table 5, the kurtosis values of ^226^Ra, ^232^Th, and ^40^K are positive values. Thus, the probability distribution of ^226^Ra, ^232^Th, and ^40^K is peaked. Table 5 also depicts the standard deviation (SD) values are less than the mean values of ^226^Ra, ^232^Th, and ^40^K activity concentrations, which reveals the uniformity in the high-level of the most results of ^226^Ra, ^232^Th, and ^40^K activity concentrations in the black sand. Table 5 displays the coefficient variance (CV, %) of ^226^Ra, ^232^Th, and ^40^K activity concentrations are moderate values (51%, 65% and 34% for ^226^Ra, ^232^Th, and ^40^K, respectively). This shows the beach black sand contains the host radioactive minerals. It can be concluded that the high activity concentrations for some radionuclides are mainly attributed to the presence of radioactive zircon, monazite, thorite and uranothorite.

**Table 5:** Descriptive statistics of data corresponding to the activity of radionuclides.

	N	Mean	SD	Min	Max	Skewness	Kurtosis	CV, %
Ra-226	40	19.1	9.7	11.1	44.4	1.1	0.4	51
Th-232	40	14.7	9.5	4.0	40.4	1.4	1.5	65
K-40	40	211.0	71.3	65.7	391.3	0.4	0.3	34

Table 6 offers the normality test of the distribution of ^226^Ra, ^232^Th, and ^40^K activity according to the Kolmogorov–Smirnov (KS) test. According to KS test, the distribution of ^226^Ra, and ^232^Th activity concentrations are non-normal. Where the distribution of ^40^K activity concentration is normal.

**Table 6:** Results of normality tests.

Radionuclide	Kolmogorov–Smirnov*		
	DF	Statistic	Asymp. Sig. (2 tail)
^226^Ra	12	0.30	0.001
^232^Th	12	0.23	0.021
^40^K	12	0.09	0.959

*Asymp. sig*. asymptotic significance level, *DF* degrees of freedom. *Lilliefors significance correction.

The second statistical analysis is the Pearson correlation (Table 7). Table 7 displays the weak correlations between ^226^Ra, and the other radionuclides ^232^Th, and ^40^K. In addition to the weak correlation between ^226^Ra and ^232^Th, with the RHI. Where a strong correlation between ^40^K and the RHI. This is due to the existence K-feldspare minerals in a light fraction of the beach black sand with a high ratio.

**Table 7:** The concentrations of radionuclides ^226^Ra, ^232^Th, ^40^K and the RHI.

	Ra-226	Th-232	K-40	D_air_	AED	ELCR
Ra-226	1					
Th-232	0.24	1				
K-40	− 0.13	0.17	1			
D_air_	0.03	0.33	0.98	1		
AED	0.03	0.33	0.98	1	1	
ELCR	0.03	0.33	0.98	1	1	1

**Figure 19:**
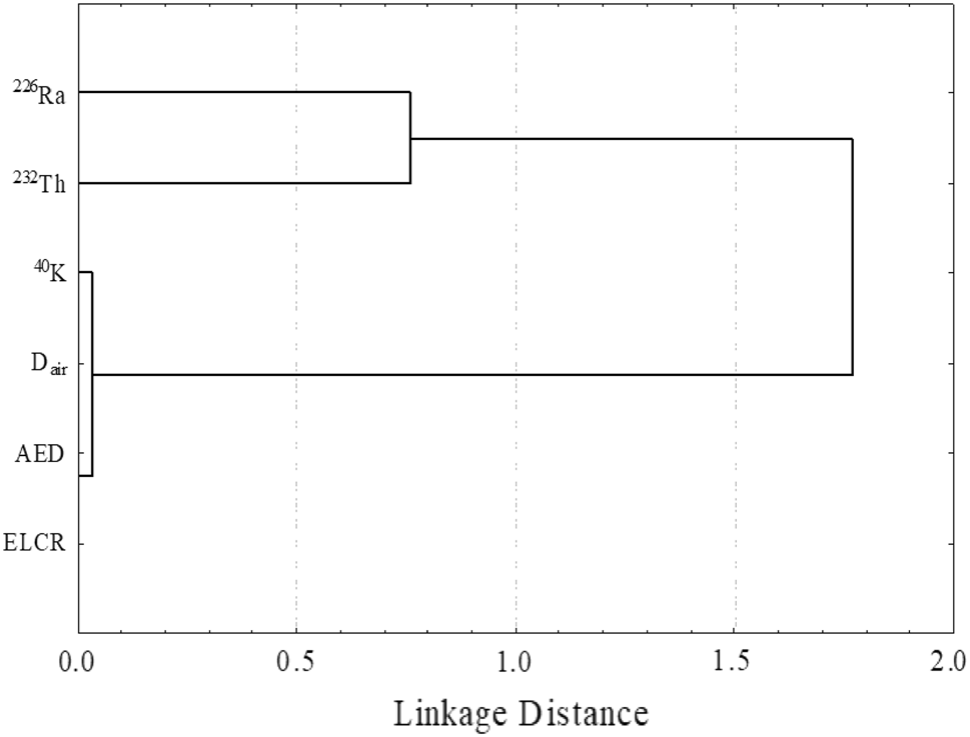
linkage between different statistical radiological hazard indices among the studied samples.

The next statistical analysis is the hierarchical cluster analysis (HCA). The HCA of the radionuclide’s activity concentrations and the RHI is presented in Fig. 19. Figure 19 reveals the dendrogram of HCA is divided into two main clusters. The first cluster includes ^226^Ra and ^232^Th. this refers to the ^226^Ra and ^232^Th are derived from the natural radioactive chains. Where the second cluster contains the ^40^K and the RHI. The reason of the correlation is stated above, which is due to the presence of K-minerals like K-feldspar in the beach black sand.

**Figure 20:**
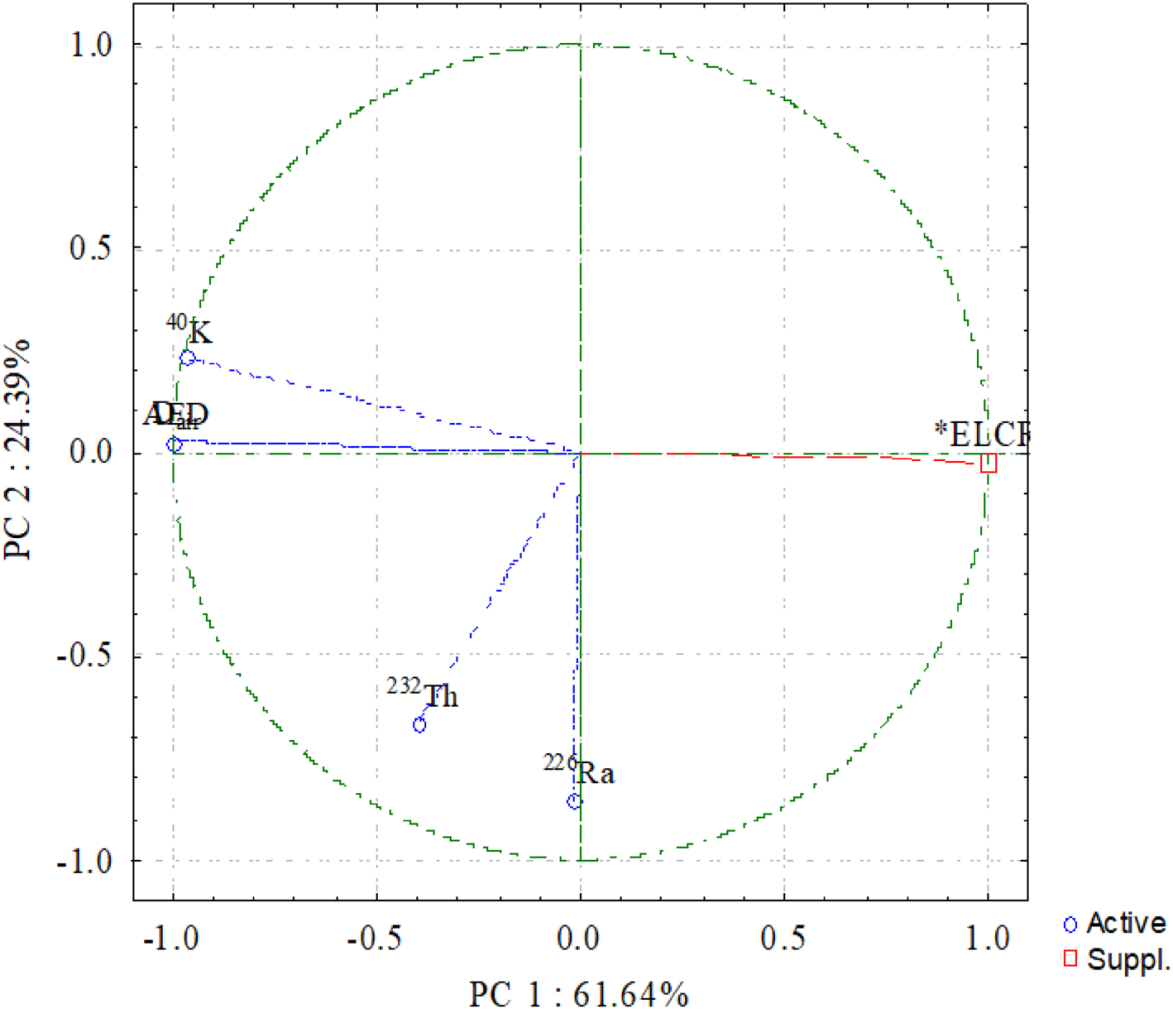
Principal component analysis (PCA) of radionuclides and radiological parameters.

The principal component analysis (PCA) is the other statistical analysis. Figure 20 confirms the RHI is linked to the K-minerals like K-feldspar in the beach black sand.

The strong positive loading of ^40^K activity concentration distinguishes the PC1, which represents 61.64% of the total variation explained. According to the positive loading of ^226^Ra and ^232^Th, the PC2 was recorded as explaining 24.39% of the overall variance. Therefore, in all beach sand in the investigated coastal zone, the ^40^K is the main emitting gamma source. Additionally, as was already noted, the mineral analysis verified the presence of the radioactively-bearing mineral monazite in the examined beach black sand. The PCA agrees with the Pearson correlation analysis and cluster analysis.

## Conclusion

This study assesses the natural radioactivity and associated hazards of stream sediments of the Baltim–El Burullus coastal plain which are it can be used in various industrial applications. The higher radioactivity in the studied stream sediments is due to several factors, such as some uranium–thorium-bearing accessory minerals such as zircon, monazite and uranothorite. The obtained results illustrated that the ^226^Ra, ^232^Th, and ^40^K activity concentrations are 19.1 ± 9.73, 14.7 ± 9.53 and 211 ± 71.34 Bq kg^−1^, respectively, lower than the reported worldwide limit 33, 45 and 412 Bq kg^−1^. Moreover, the mean value of AED of the stream sediments was 0.03 mSv year^−1^, respectively, which lower approximately two times than the worldwide values. Therefore, the studied stream sediments is safe for use in various industrial application and infrastructure fields. The multivariate statistical analysis suggests that the estimated radiological factors are related to the activity concentrations of the investigated radionuclides in the stream sediments.

## Data Availability

All data generated or analyzed during this study are included in this published article.
